# Genotyping and surveillance for scrapie in Finnish sheep

**DOI:** 10.1186/1746-6148-8-122

**Published:** 2012-07-25

**Authors:** Maria Hautaniemi, Hannele Tapiovaara, Sirkka-Liisa Korpenfelt, Liisa Sihvonen

**Affiliations:** 1 Research Department/Veterinary Virology, Finnish Food Safety Authority Evira, Mustialankatu 3, FI-00790, Helsinki, Finland; 2 Department of Veterinary Biosciences/Faculty of Veterinary Medicine, University of Helsinki, Agnes Sjöbergin katu 2, FI-00014, Helsinki, Finland

## Abstract

**Background:**

The progression of scrapie is known to be influenced by the amino acid polymorphisms of the host prion protein (PrP) gene. There is no breeding programme for TSE resistance in sheep in Finland, but a scrapie control programme has been in place since 1995.

In this study we have analysed PrP genotypes of total of 928 purebred and crossbred sheep together with the data of scrapie survey carried out in Finland during 2002–2008 in order to gain knowledge of the genotype distribution and scrapie prevalence in Finnish sheep.

**Results:**

The ARQ/ARQ genotype was the most common genotype in all breeds studied. ARR allele frequency was less than 12% in purebred Finnish sheep and in most genotypes heterozygous for ARR, the second allele was ARQ. The VRQ allele was not detected in the Grey race sheep of Kainuu or in the Aland sheep, and it was present in less than 6% of the Finnish Landrace sheep. Leucine was the most prominent amino acid found in codon 141. In addition, one novel prion dimorphisms of Q220L was detected. During the scrapie survey of over 15 000 sheep in 2002–2008, no classical scrapie cases and only five atypical scrapie cases were detected.

**Conclusions:**

The results indicate that the Finnish sheep populations have genetically little resistance to classical scrapie, but no classical scrapie was detected during an extensive survey in 2002–2008. However, five atypical scrapie cases emerged; thus, the disease is present in the Finnish sheep population at a low level.

## Background

Scrapie is a transmissible, naturally occurring neurological disease of sheep and goats. It is characterized by a long incubation period and gradual degeneration of the central nervous system, invariably leading to animal’s death. A number of clinical signs, e.g. pruritus, weight loss, salivation, nervousness, altered behaviour and ataxia, are associated with the disease. Scrapie has been recognized as a clinical disorder of sheep worldwide for more than 270 years [1,2]. The assumed aetiological agent of scrapie is a protease-resistant isoform of the host-encoded prion protein (PrP^Sc^) [3]. Although scrapie is an infectious disease, certain amino acid polymorphisms in the sheep prion protein (PrP) affect sheep’s susceptibility to the disease [4-6]. Especially valine or alanine at position 136 combined with arginine and glutamine at positions 154 and 171, respectively, are associated to susceptibility to classical scrapie. The greatest risk for both natural and experimental scrapie is associated with genotypes encoding V_136_R_154_Q_171_ (VRQ). The second greatest risk is associated with the A_136_R_154_Q_171_ (ARQ) homozygous genotype; moreover, it has been indicated that this genotype is associated with increased scrapie susceptibility in breeds where the VRQ allele is rare or absent [6-8]. The A_136_R_154_R_171_ allele (ARR) has generally been shown to slow down the progression of both natural and experimental scrapie in sheep [4,6,9]. In addition to polymorphisms at codons 136, 154 and 171 of the prion gene, many other polymorphisms have been described in sheep [10,11].

As the result of the increased scrapie surveillance, a number of atypical cases have been identified in many European countries [12]. Genetic susceptibility to atypical scrapie is reported to be associated with different genotypes than in classical scrapie. Atypical cases are generally found in animals carrying the A_136_H_154_Q_171_ (AHQ) or ARQ allele, and also sheep with the ARR/ARR genotype has been shown to be infected naturally with atypical scrapie. Moreover, phenylalanine at codon 141 has been associated to atypical scrapie [13-15].

The Finnish sheep population consists of about 55 000 ewes aged over 12 months living on 1000 farms. The average number of ewes on a farm is approximately 55, and only 150 farms have more than 150 ewes in a flock. Majority of the sheep are of Finnish Landrace sheep, but a small proportion of other breeds exist, including the Aland sheep, the Grey race sheep of Kainuu and Texel. Scrapie is mandatory notifiable disease in Finland and a scrapie control program was established in 1995. There is no breeding programme for TSE resistance in sheep in Finland. This paper describes the results of a scrapie survey and prion protein genotypes of the main breeds of sheep in Finland studied during 2002–2008 in conjunction with the epidemiology of Finnish atypical scrapie cases.

## Results

### PrP genotypes of Finnish pure- and crossbred sheep

The frequencies of PrP genotypes of altogether 928 purebred and crossbred Finnish sheep regarding the codons 136, 141, 154 and 171 are presented in Table 1, and the frequencies of different alleles of Finnish sheep breeds are summarized in Table 2.

**Table 1:** PrP genotypes of Finnish sheep

**PrP genotype**	**Breed**									
	**Finnish landrace**		**Grey race sheep of Kainuu**		**Aland sheep**		**Texel**		**Crossbred**	
	**n**	**%**	**n**	**%**	**n**	**%**	**n**	**%**	**n**	**%**
ARQ/ARQ	161	68.8	40	83.3	27	48.2	24	33.8	279	53.7
ARR/ARQ	37	15.9	8	16.7	13	23.2	22	31.0	132	25.3
ARR/ARR	3	1.3					1	1.4	26	5.0
ARR/VRQ									2	0.4
ARR/AHQ									1	0.2
ARR/ARH	1	0.4					1	1.4	5	1.0
ARH/ARH							2	2.8	2	0.4
ARQ/VRQ	24	10.3					2	2.8	23	4.4
ARQ/ARH	2	0.9					13	18.3	23	4.4
AHQ/AHQ					5	8.9			5	1.0
ARH/VRQ									1	0.2
ARQ/AHQ	1	0.4			11	19.6	6	8.5	18	3.5
VRQ/VRQ	1	0.4							1	0.2
AFRQ/AFRQ	1	0.4								
AFRQ/ARQ	1	0.4							3	0.6
Total	232	100.0	48	100.0	56	100.0	71	100.0	521	100.0

n = number of animals.

**Table 2:** PrP allele frequences of purebred sheep

**Allele**	**Breed**			
	**Finnish Landrace**	**Grey race sheep of Kainuu**	**Aland sheep**	**Texel**
	**n = 464**	**n = 96**	**n = 112**	**n = 144**
ARQ	83.4	91.7	69.6	64.1
ARR	9.5	8.3	11.6	17.6
VRQ	5.6	0	0	1.4
AHQ	0.2	0	18.8	4.2
ARH	0.6	0	0	12.7
AF141RQ	0.6	0	0	0
Total	100.0	100.0	100.0	100.0

n = number of alleles.

ARQ and ARR alleles were present in all the sheep breeds analysed, the ARQ being the dominant allele (64.1–91.7%, Table 2) and ARQ/ARQ being the prevailing genotype (Table 1). Sheep with the ARR/ARQ genotype was the second largest group in all breeds studied (15.9–25.3%). VRQ/VRQ genotype was extremely rare; only 0.4% and 0.2% of Finnish Landrace and crossbred sheep, respectively, were shown to have this genotype. AHQ allele frequency was found to be notably higher in the Aland sheep (18.8%) compared to Finnish Landrace sheep (0.2%), Grey race sheep of Kainuu (0%) and Texel (4.2%) (Table 2).

Of the sheep breeds studied, only two Finnish Landrace was shown to carry F at codon 141 (Tables 1 and 2) and three crossbred sheep had the F_141_ polymorphism linked with one ARQ haplotype (hereafter AFRQ) (Table 1).

In addition to the L141F polymorphism, some other polymorphisms were detected in the sheep genotyped by sequencing the complete PrP open reading frame during 2002–2003. These polymorphisms are presented as a wild type codon mutation e.g. R151C in Table 3. All sheep either homozygous or heterozygous for the polymorphisms carried at least one ARQ haplotype. Altogether eight dimorphisms out of 410 sheep samples sequenced in full, specifically G89S, M137T, H143R, R151C, R211Q, Q220L, E224K and P241S, were detected. All of them except Q220L have been described before [5,10,16-18]. The most common dimorphisms detected in Finnish Landrace sheep were R151C and E224K with allele frequencies 6.6% and 5.8%, respectively. R151C was also the most common dimorphism detected in Texel sheep (allele frequency 6.6%). The frequency of the dimorphism E224K was relatively high (18.8%) in Grey race sheep of Kainuu.

**Table 3:** Polymorphisms of the PrP gene

**Polymorphism**	**Breed**									
	**Finnish Landrace**		**Grey race sheep of Kainuu**		**Aland sheep**		**Texel**		**Crossbred**	
	**n = 258**	**%**	**n = 96**	**%**	**n = 98**	**%**	**n = 122**	**%**	**n = 246**	**%**
G89S, E224K	2	0.8								
M137T							1	0.8	8	3.3
H143R					1	1.0				
R151C	17	6.6					8	6.6	7	2.8
R151C, E224K	1	0.4								
R211Q, Q220L									1	0.4
E224K	15	5.8	18	18.8			1	0.8	7	2.8
P241S							1	0.8	3	1.2

n = number of alleles.

### Surveillance of sheep for the presence of scrapie

A total of 15 804 sheep were tested for scrapie in Finland during 2002–2008. No classical scrapie was found, whereas five cases of atypical scrapie were discovered, corresponding to a frequency of 0.03% of all sheep studied (Table 4). The first of these cases occurred in 2004 and was detected in the brainstem of a 9-year-old Finnish Landrace sheep first as a weak positive result in Prionics Western blot test and subsequently with BioRad TeSeE test. The other four cases were detected with BioRad TeSeE test either in the brainstem only or in both the brainstem and cerebellum (Table 5). All the cases were confirmed as atypical scrapie with SAF immunoblot [19] and/or BioRad TeSeE Western blot test at the time of their occurrence (results not shown). In addition, three of the positive samples were confirmed as atypical scrapie in the Norwegian TSE reference laboratory (National Veterinary Institute, Norway) and one of them was also confirmed in Animal Health and Veterinary Laboratories Agency (AHVLA, Weybridge).

**Table 4:** Samples tested for scrapie in Finland

**Year**	**Healthy/**^**1**^**Positive**	**Fallen stock/Positive**	**Emergency slaughtered/Positive**	**Clinical signs ante mortem/Positive**	**TSE Suspects/Positive**	**Eradication/Positive**	**Scrapie positive**	**Strain**^**2**^	**Percentage of positive in fallen/healthy**
2002	2053/0	331/0	15/0	3/0	0/0	16/0	0		
2003	1989/0	683/0	0/0	2/0	0/0	0/0	0		
2004	498/0	800/1	2/0	3/0	2/0	37/0	1	Atypical	0.12/0
2005	392/0	899/1	1/0	1/0	1/0	43/0	1	Atypical	0.11/0
2006	2784/1	916/1	0/0	0/0	0/0	134/0	2	Atypical	0.11/0.04
2007	1944/0	1073/1	2/0	1/0	0/0	10/0	1	Atypical	0.09/0
2008	0/0	1163/0	0/0	0/0	1/0	0/0	0		
Total	9660/1	5865/4	20/0	10/0	4/0	240/0	5		

^1^The number after "/" of each group indicates the number of scrapie positive in that group.

^2^Atypical scrapie [20].

**Table 5:** Atypical scrapie cases in Finland

**Year**	**Sample no**	**Sample material**	**Age in years**	**Race**		**Genotype**
2004	1/2004	brain stem	9	Finnish Landrace	Fallen stock	ALRQ/ALRQ
2005	1/2005	brain stem	10	Finnish Landrace	Fallen stock	NA
2006	1/2006	brain stem and cerebellum	4	Finnish Landrace	Fallen stock	ALRH/ALHQ
2006	2/2006	brain stem and cerebellum	unknown	Finnish Landrace	Healthy	ALRQ/ALRQ
2007	1/2007	brain stem	2,3	Finnish Landrace	Fallen stock	AFRQ/AFRQ

NA = not applicable due to poor sample quality.

Background information and genotypes of the atypical scrapie cases are shown in Table 5. The annual frequency of atypical scrapie in fallen stock sheep varied between 0 and 0.12%. Amongst healthy sheep, only one case was found in 2006, corresponding to a frequency of 0.04% (Table 4). None of the atypical scrapie cases were reported to have clinical signs.

## Discussion

Sheep husbandry is a minor agricultural industry in Finland. The Finnish sheep population is small and the majority of the animals are old Finnish breeds, the most important of which is Finnish Landrace. In addition, a small proportion of other breeds exist, including Aland sheep, the Grey race sheep of Kainuu and Texel.

Our results provide the first data of PrP genotypes of these main landraces, both purebred and crossbred sheep, together with a small proportion of Texel sheep. The predominant allele in the Finnish breeds was ARQ (69.6–91.7%). ARQ is regarded as a wild type allele; it has also been found at a high frequency in old sheep breeds of Iceland [17,21,22]. Like Icelandic sheep and native Austrian mountain sheep races, the Finnish Landrace is well adapted to harsh Nordic climate and aseasonal breeding. In addition, in these old breeds, only a few alleles dominate; we found altogether 13 genotypes in Finnish breeds, but nine of them were present in less than 10% of the sheep studied. Genotypes of Grey race sheep of Kainuu consisted of only ARQ and ARR alleles, the allelic distribution of Aland sheep was ARQ, AHQ and ARR, whereas Finnish Landrace sheep exhibited all five commonly observed alleles ARQ, ARR, VRQ, ARH and AHQ. In terms of assessing clinical scrapie risk with the National Scrapie Plan for Great Britain [23] categorization, where R1 correspond to very low classical scrapie risk and R5 the highest classical scrapie risk, 71.6% of Finnish Landrace sheep, 76.8% of Aland sheep and 83.3% of Grey race sheep of Kainuu would fall into risk group R3. Compared with breeds in Great Britain, the high ARQ and notable AHQ allele frequency of Aland sheep corresponds to the allele distribution of Bluefaced Leicester sheep, whereas the Grey race sheep of Kainuu with an absent VRQ allele resembles Suffolk sheep, both of which are reported to be highly susceptible to classical scrapie [7,8,24].

Our results indicate that Finnish sheep have genetically little resistance to classical scrapie. However, classical scrapie has never been detected in sheep in Finland despite intensive surveys. The likely explanation for this is non-existent or low infection pressure of classical scrapie due to Finland’s limited and strict import policy of live sheep intended to prevent the introduction of diseases. Prior to 1995, only a small number of sheep from Denmark and Sweden were imported [25], and after 1995 only a few sheep have been imported to nine farms. All of the imported sheep after 1995 were from countries where the scrapie control programme is in place. The two farms that imported sheep from countries outside the scrapie programme are under strict control. Our results support the view that development of scrapie requires both susceptible genotype and sheep’s exposure to an infecting scrapie strain [8,26].

In the intensive active surveillance for scrapie in sheep in Finland during 2002–2008, no classical scrapie was found, but five atypical scrapie cases were detected. It is noteworthy that we detected the first atypical scrapie case by using Prionics Western blot test until it became general knowledge that atypical scrapie is sensitive to the amount of proteinase K used by the method and could thus go unnoticed [20,27]. Despite the higher concentrations of PrPSc in the cerebellum than in the obex [28], in three out of five cases the diagnosis was made from the obex, as the cerebellum was not available.

The genotype was obtained from four out of five atypical cases. Atypical scrapie is most often associated to the ARQ allele, and contrary to classical scrapie, to the AHQ and ARR alleles. In addition, sheep with atypical scrapie have often been found to carry phenylalanine (F), instead of leucine (L), at codon 141, usually in ARQ homozygotes [13,14,20]. In our study one of the five atypical scrapie sheep had the AFRQ genotype, although the AFRQ allele was rare in the studied sheep population (Tables 1 and 2). These results support the association of AFRQ with atypical scrapie. Furthermore, it has been reported that the genotypes of the youngest animals infected with Nor98 atypical scrapie were AFRQ/AFRQ, AFRQ/ALHQ or ALHQ/ALHQ, whereas atypical scrapie sheep with the ALRQ/ALRQ genotype were clearly older [13]. The Finnish atypical scrapie sheep with the AFRQ genotype was slightly over two years old, the sheep with the ALHQ allele was four years old and the sheep with the ALRQ genotype was nine years old, but because all of them were fallen stock without any known clinical signs it cannot be concluded if the age of the Finnish cases is associated to the onset of atypical scrapie.

In addition to the known atypical scrapie-associated polymorphism L141F, some other previously characterized and novel polymorphisms linked to the ARQ allele were found in the Finnish sheep population. The most frequent polymorphisms present in Finnish Landrace were R151C and E224K (6.6 and 5.8% of sheep studied, respectively), both of which have been described before [17,18,29]. Variation in ovine PrP gene is common, but an association of these polymorphisms with scrapie is mostly unknown [11]. However, partial resistance with natural scrapie has been associated to R151C in one flock of Icelandic sheep [17] and to M112T in Japanese Suffolk and Corriedale sheep [30]. The importance of the relatively high frequency of R151C in Finnish Landrace is unknown. The dimorphism of E224K found in Finnish Landrace (5.8%) and Grey race sheep of Kainuu (18.8%) has recently been described in Polish sheep breeds [18]. To our knowledge the study of Piestrzynska-Kajtoch et al. (2012) and this study are the only reports of E224K polymorphism so far. This might suggest that there would be a link between Finnish and Polish sheep, but this is yet to be determined. There has been no import of live sheep from Poland to Finland, but it cannot be excluded that some Finnish landrace sheep might have been exported to Poland from Finland or from other countries.

The epidemiology of atypical scrapie remains incompletely understood. The disease has been experimentally transmitted to ovinized transgenic mice and sheep with the genotype AHQ/AHQ [31,32]. This indicates that the disease could be transmitted within a flock in natural conditions, although this is yet to be established. It has also been suggested that atypical scrapie could arise spontaneously its being common to find only one atypical case per flock [12,13,15]. This suggestion is supported by the finding that atypical scrapie is usually detected throughout the country, and typically only a single positive sheep is identified in an affected flock [12,28,33]. All five Finnish atypical cases were single cases in their flock, and no epidemiological connections existed between them. Four out of the five atypical scrapie cases were originally detected in the brainstem which was somewhat surprising because it is generally known that the highest concentration of PrPSc in atypical scrapie is detected in the cerebellum. However, it has been reported that in some cases of atypical scrapie PrPSc deposition can be detected in the obex region of the brainstem [32,34]. The prevalence of atypical scrapie in Finnish sheep was similar to that in European sheep [12,15,33]. The prevalence was slightly lower in the healthy sheep population than in fallen stock animals. This might be due to the mean age of fallen sheep being higher, as the incidence of atypical scrapie has been reported to be higher in older sheep [13,35].

## Conclusions

Atypical scrapie is present in the Finnish sheep population at a low level. No classical scrapie was detected in the intensive survey conducted in 2002–2008, although the population has genetically little resistance to classical scrapie.

## Methods

### Sample collection and DNA purification

Blood samples were randomly taken from purebred and crossbred sheep flocks around Finland, altogether 720 samples during the years 2002–2008. The blood samples, as well as the brain samples described in the section below, were collected by official veterinarians as a part of Finnish scrapie surveillance programme and requirements set out in Regulation (EC) No 999/2001, Annex 2001. Therefore no experimental research on animals was carried out during this study. Sampling was arranged and samples were received by the personnel of the Evira’s TSE laboratory. In addition, we studied blood samples collected from different purebred flocks of Grey race sheep of Kainuu (48 samples), Aland sheep (45 samples), Finnish Landrace (64 samples) and Texel (51 samples) in 2002. Genotyping of scrapie-positive sheep was done from brain tissue. DNA from blood samples was extracted with the QIAamp® DNA Mini Kit (Qiagen Inc., Valencia, CA, USA) and DNA from scrapie infected brain tissue was extracted using Chelex 100 chelating resin (Bio-Rad Laboratories Inc., Valencia, CA, USA) and phenol-chloroform extraction method [36].

### PrP PCR

PrP was amplified in PCR producing 905 bp amplicon with primers F1832 5’ GGG CATTTGATGCTGACAC and F1833 5’ TGA GACACCACCACTACAG flanking the PrP coding region. Amplification was done in a 50 μl reaction mixture containing 100 ng of purified DNA, each deoxynucleotide at a concentration of 0.2 μM, 15 pmol of both primers, 10 mM Tris–HCl, pH 8.3; 50 mM KCl, 1.5 mM MgCl_2_ and 2U of AmpliTaqGold (Applied Biosystems, Foster City, CA, USA). Cycling conditions after initial denaturation at 95 °C 8 min were 30 cycles of denaturation (95 °C, 1 min), annealing (53 °C, 1 min) and extension (72 °C, 1 min).

### Genotyping

PCR products were sequenced either at the DNA Synthesis and Sequencing Laboratory, Institute of Biotechnology, University of Helsinki (2002–2003), or at EELA/Evira since 2004. The sequence of both strands of the PCR products were determined using either the PCR primers F1832 and F183 producing a sequence comprising entire PrP open reading frame, or the primers PrPR1 5’ ACATTTGCTCCACCACTCG and PrPF1 5’ AGCCACAGTCAGTGGAAC producing a sequence comprising codons 104–210 of the PrP coding region.

Sequencing reactions were run on ABI 3700 or ABI 3100-Avant genetic analysers using BigDye Terminator chemistry (Applied Biosystems). The chromatograms were checked manually with the help of programs SeqScape v2.1.1. (Applied Biosystems) or Staden Package [37] using the Suffolk sheep PrP coding region as a reference (GenBank accession no. M31313), and the polymorphisms at codons 136, 141, 154 and 171 were determined.

### Brain sampling

Altogether samples from 15 804 sheep were studied for the presence of scrapie during the years 2002–2008. The sheep brain stem was removed with a sampling spoon. Detection of atypical scrapie cases in Europe led to changes in the brain sampling protocol, and hence, since 2004, a second sample containing cerebellum was collected when possible.

### TSE testing

The brain samples collected from healthy sheep were analysed with Bio-Rad TeSeE® test (Bio-Rad, Nazareth, Belgium). Brain samples from fallen stock, emergency-slaughtered and sick sheep were analysed with Prionics® Check WESTERN (Prionics, Schlieren-Zurich, Switzerland) from 1.1.2002 to 31.12.2004, and starting from 1.1.2004 with the Bio-Rad TeSeE® test. The brain samples were homogenized, treated with Proteinase K and analysed in rapid tests according to manufacturers´ instructions. Positive scrapie cases were confirmed by SAF immunoblot according to standardized methodology [19] and/or by BioRad TeSeE® Western blot test (Bio-Rad, Nazareth, Belgium) according to manufacturers´ instructions.

## Competing interests

This work was conducted in and supported by Finnish Food Safety Authority Evira. The genotyping and survey for scrapie were carried out in Evira. MH, HT and SLK were and are full time employees in Evira. LS is partly employed as research director by Evira and partly as professor of veterinary virology by University of Helsinki. Evira has supported financially the study.

## Authors’ contributions

MH participated in the design of the study, set up the genotyping methods and analysed the data. She also participated in carrying out the genotyping and TSE surveys and wrote the manuscript. HT conceived and participated in the design of the study, participated in carrying out the TSE and genotyping surveys. She had an important input into and participated in writing the manuscript. SLK participated in carrying out the TSE survey and commented the manuscript. LS conceived and participated in the design of the study, coordinated the work and helped in writing the manuscript. All authors read and approved the final manuscript.

## References

[B1] Prusiner SB Prions Proc Natl Acad Sci U S A 1998 95 23 13363 13383 10.1073/pnas.95.23.13363 9811807 PMC33918

[B2] Detwiler LA Baylis M The epidemiology of scrapie Rev Sci Tech 2003 22 1 121 143 12793776 10.20506/rst.22.1.1386

[B3] Diener TO McKinley MP Prusiner SB Viroids and prions Proc Natl Acad Sci U S A 1982 79 17 5220 5224 10.1073/pnas.79.17.5220 6813855 PMC346867

[B4] Goldmann W Hunter N Smith G Foster J Hope J PrP genotype and agent effects in scrapie: change in allelic interaction with different isolates of agent in sheep, a natural host of scrapie J Gen Virol 1994 75 Pt 5 989 995 7909834 10.1099/0022-1317-75-5-989

[B5] Belt PB Muileman IH Schreuder BE Bos-de Ruijter J Gielkens AL Smits MA Identification of five allelic variants of the sheep PrP gene and their association with natural scrapie J Gen Virol 1995 76 Pt 3 509 517 7897344 10.1099/0022-1317-76-3-509

[B6] Baylis M Chihota C Stevenson E Goldmann W Smith A Sivam K Tongue S Gravenor MB Risk of scrapie in British sheep of different prion protein genotype J Gen Virol 2004 85 Pt 9 2735 2740 15302967 10.1099/vir.0.79876-0

[B7] Westaway D Zuliani V Cooper CM Da Costa M Neuman S Jenny AL Detwiler L Prusiner SB Homozygosity for prion protein alleles encoding glutamine-171 renders sheep susceptible to natural scrapie Genes Dev 1994 8 8 959 969 10.1101/gad.8.8.959 7926780

[B8] Hunter N Moore L Hosie BD Dingwall WS Greig A Association between natural scrapie and PrP genotype in a flock of Suffolk sheep in Scotland Vet Rec 1997 140 3 59 63 10.1136/vr.140.3.59 9023905

[B9] Groschup MH Lacroux C Buschmann A Luhken G Mathey J Eiden M Lugan S Hoffmann C Espinosa JC Baron T Torres JM Erhardt G Andreoletti O Classic scrapie in sheep with the ARR/ARR prion genotype in Germany and France Emerg Infect Dis 2007 13 8 1201 1207 10.3201/eid1308.070077 17953092 PMC2828083

[B10] Heaton MP Leymaster KA Freking BA Hawk DA Smith TP Keele JW Snelling WM Fox JM Chitko-McKown CG Laegreid WW Prion gene sequence variation within diverse groups of U.S. sheep, beef cattle, and deer Mamm Genome 2003 14 11 765 777 10.1007/s00335-003-2283-y 14722726

[B11] Goldmann W PrP genetics in ruminant transmissible spongiform encephalopathies Vet Res 2008 39 4 30 10.1051/vetres:2008010 18284908

[B12] Fediaevsky A Tongue SC Noremark M Calavas D Ru G Hopp P A descriptive study of the prevalence of atypical and classical scrapie in sheep in 20 European countries BMC Vet Res 2008 4 19 10.1186/1746-6148-4-19 18544152 PMC2442063

[B13] Moum T Olsaker I Hopp P Moldal T Valheim M Moum T Benestad SL Polymorphisms at codons 141 and 154 in the ovine prion protein gene are associated with scrapie Nor98 cases J Gen Virol 2005 86 Pt 1 231 235 15604451 10.1099/vir.0.80437-0

[B14] Saunders GC Cawthraw S Mountjoy SJ Hope J Windl O PrP genotypes of atypical scrapie cases in Great Britain J Gen Virol 2006 87 Pt 11 3141 3149 17030846 10.1099/vir.0.81779-0

[B15] Luhken G Buschmann A Brandt H Eiden M Groschup MH Erhardt G Epidemiological and genetical differences between classical and atypical scrapie cases Vet Res 2007 38 1 65 80 10.1051/vetres:2006046 17156738

[B16] Bossers A Schreuder BE Muileman IH Belt PB Smits MA PrP genotype contributes to determining survival times of sheep with natural scrapie J Gen Virol 1996 77 Pt 10 2669 2673 8887505 10.1099/0022-1317-77-10-2669

[B17] Thorgeirsdottir S Sigurdarson S Thorisson HM Georgsson G Palsdottir A J Gen Virol 1999 80 Pt 9 2527 2534 10501510 10.1099/0022-1317-80-9-2527

[B18] Piestrzynska-Kajtoch A Gurgul A Polak MP Smolucha G Zmudzinski JF Rejduch B Characterization of PRNP and SPRN coding regions from atypical scrapie cases diagnosed in Poland Mol Biol Rep 2012 39 3 2575 2583 10.1007/s11033-011-1010-0 21674189

[B19] OIE Bovine spongiform encephalopathy, Chapter 2.3.13 Manual of diagnostic tests and vaccines for terrestrial animals (mammals, birds and bees) 2004 Office International des Épizooties, Paris, France

[B20] Benestad SL Sarradin P Thu B Schonheit J Tranulis MA Bratberg B Cases of scrapie with unusual features in Norway and designation of a new type, Nor98 Vet Rec 2003 153 7 202 208 10.1136/vr.153.7.202 12956297

[B21] Sipos W Kraus M Schmoll F Achmann R Baumgartner W PrP genotyping of Austrian sheep breeds J Vet Med A Physiol Pathol Clin Med 2002 49 8 415 418 10.1046/j.1439-0442.2002.00472.x 12450189

[B22] Tsunoda K Namikawa T Sato K Hasnath MA Nyunt MM Rajbandary HB Loc CB Zanchiv T Chang H Sun W Dorji T Prion protein polymorphisms and estimation of risk of scrapie in East Asian sheep Biochem Genet 2010 48 1–2 13 25 19731007 10.1007/s10528-009-9287-6

[B23] Anonymous National Scrapie Plan for Great Britain 2006 http://adlib.everysite.co.uk/resources/000/054/063/NSP_english.pdf

[B24] Dawson M Hoinville LJ Hosie BD Hunter N Guidance on the use of PrP genotyping as an aid to the control of clinical scrapie. Scrapie Information Group Vet Rec 1998 142 23 623 625 9650232

[B25] Sihvonen L Hirvela-Koski V Nuotio L Kokkonen UM Serological survey and epidemiological investigation of maedi-visna in sheep in Finland Vet Microbiol 1999 65 4 265 270 10.1016/S0378-1135(98)00312-5 10223325

[B26] Hunter N Cairns D Foster JD Smith G Goldmann W Donnelly K Is scrapie solely a genetic disease? Nature 1997 386 6621 137 9062185 10.1038/386137a0

[B27] Buschmann A Biacabe AG Ziegler U Bencsik A Madec JY Erhardt G Luhken G Baron T Groschup MH Atypical scrapie cases in Germany and France are identified by discrepant reaction patterns in BSE rapid tests J Virol Methods 2004 117 1 27 36 10.1016/j.jviromet.2003.11.017 15019257

[B28] Benestad SL Arsac JN Goldmann W Noremark M Atypical/Nor98 scrapie: properties of the agent, genetics, and epidemiology Vet Res 2008 39 4 19 10.1051/vetres:2007056 18187032

[B29] Goldmann W Baylis M Chihota C Stevenson E Hunter N Frequencies of PrP gene haplotypes in British sheep flocks and the implications for breeding programmes J Appl Microbiol 2005 98 6 1294 1302 10.1111/j.1365-2672.2005.02568.x 15916643

[B30] Ikeda T Horiuchi M Ishiguro N Muramatsu Y Kai-Uwe GD Shinagawa M Amino acid polymorphisms of PrP with reference to onset of scrapie in Suffolk and Corriedale sheep in Japan J Gen Virol 1995 76 Pt 10 2577 2581 7595361 10.1099/0022-1317-76-10-2577

[B31] Le Dur A Beringue V Andreoletti O Reine F Lai TL Baron T Bratberg B Vilotte JL Sarradin P Benestad SL Laude H A newly identified type of scrapie agent can naturally infect sheep with resistant PrP genotypes Proc Natl Acad Sci U S A 2005 102 44 16031 16036 10.1073/pnas.0502296102 16239348 PMC1276041

[B32] Simmons MM Konold T Simmons HA Spencer YI Lockey R Spiropoulos J Everitt S Clifford D Experimental transmission of atypical scrapie to sheep BMC Vet Res 2007 3 20 10.1186/1746-6148-3-20 17725818 PMC2025597

[B33] Fediaevsky A Maurella C Noremark M Ingravalle F Thorgeirsdottir S Orge L Poizat R Hautaniemi M Liam B Calavas D Ru G Hopp P The prevalence of atypical scrapie in sheep from positive flocks is not higher than in the general sheep population in 11 European countries BMC Vet Res 2010 6 9 10.1186/1746-6148-6-9 20137097 PMC2832631

[B34] Gavier-Widen D Stack MJ Baron T Balachandran A Simmons M Diagnosis of transmissible spongiform encephalopathies in animals: a review J Vet Diagn Invest 2005 17 6 509 527 10.1177/104063870501700601 16475509

[B35] Gavier-Widen D Noremark M Benestad S Simmons M Renstrom L Bratberg B Elvander M af Segerstad CH Recognition of the Nor98 variant of scrapie in the Swedish sheep population J Vet Diagn Invest 2004 16 6 562 567 10.1177/104063870401600611 15586572

[B36] Sambrook S Fritsch EF Maniatis T Ford N, Nolan C, Ferguson M Commonly used techniques in molecular cloning Molecular cloning: a laboratory manual 1989 2 Cold Spring Harbor Laboratory Press, New York

[B37] Staden R Beal KF Bonfield JK The Staden package, 1998 Methods Mol Biol 2000 132 115 130 10547834 10.1385/1-59259-192-2:115

